# Dynamic Leadership Mechanism in Homing Pigeon Flocks

**DOI:** 10.3390/biomimetics9020088

**Published:** 2024-02-01

**Authors:** Lin Xie, Xiangyin Zhang

**Affiliations:** 1 Faculty of Information Technology, Beijing University of Technology, Beijing 100124, China; xielin0729@163.com; 2 Engineering Research Center of Digital Community, Ministry of Education, Beijing 100124, China

**Keywords:** homing flight, dynamic leadership, acceleration, swarm motion

## Abstract

In recent years, an increasing number of studies have focused on exploring the principles and mechanisms underlying the emergence of collective intelligence in biological populations, aiming to provide insights for human society and the engineering field. Pigeon flock behavior garners significant attention as a subject of study. Collective homing flight is a commonly observed behavioral pattern in pigeon flocks. The study analyzes GPS data during the homing process and utilizes acceleration information, which better reflects the flock’s movement tendencies during turns, to describe the leadership relationships within the group. By examining the evolution of acceleration during turning, the study unveils a dynamic leadership mechanism before and after turns, employing a more intricate dynamic model to depict the flock’s motion. Specifically, during stable flight, pigeon flocks tend to rely on fixed leaders to guide homing flight, whereas during turns, individuals positioned in the direction of the flock’s turn experience a notable increase in their leadership status. These findings suggest the existence of a dynamic leadership mechanism within pigeon flocks, enabling adaptability and stability under diverse flight conditions. From an engineering perspective, this leadership mechanism may offer novel insights for coordinating industrial multi-robot systems and controlling drone formations.

## 1. Introduction

Cooperative behavior is frequently observed in biological systems at both micro and macro levels in the natural world. Bacterial colonies [1], fish schools [2], bird flocks [3], and mammal herds [4,5,6] all exhibit collective behavior. The study of collective motion emerged in the 1990s with the advent of computer simulations. In 1995, Vicsek et al. put forth the widely recognized flocking model to study the emergence of self-organized motion in particle systems with biological motivation and interactions. This model, known as the Vicsek model (VM) [7], can simulate ordered behavior in bird flocks. Additionally, in 2002, Couzin et al. [8] presented a self-organizing model to investigate the spatial dynamics of animal groups, including fish schools and bird flocks, in three-dimensional space.

The question of how individual differences shape animal groups, affect the flow of information and determine levels of decision-making authority is a crucial aspect in the study of collective behavior. Nagy et al. [9] identified a well-defined hierarchical structure among bird flock members based on data regarding dominant pairwise interactions, indicating that stratified organization in collective flight may be more efficient than egalitarian organization. Del Mar Delgado et al. [10] investigated the influence of individual memory and information transmission on the foraging success of a group, revealing their facilitation of coordinated behavior within the group in complex environments. Sasaki et al. [11] explored the connection between individual “personality” and leadership roles in collective movement, discovering that pigeons displaying higher levels of exploratory behavior (i.e., “bold” individuals) were more likely to hold elevated positions in the leadership hierarchy and exert greater influence on the direction of group movement. In the context of the leadership and flight mechanics of goose family groups during spring migration, previous research has indicated that the leadership role was typically fixed and did not incur excessive costs [12]. Chen et al. [13] revealed the existence of a joint-connected topology within pigeon flocks, which effectively reduces communication and information processing demands while ensuring the group’s agility and stability. At the same time, some scholars have revealed the hidden interaction mechanism of three groups of pigeons flying freely based on the information theory causal inference method, which is local interaction [14].

The study and analysis of biological collective behavior mechanisms have broad applications and significance in many fields, and many studies have been inspired by animal collective behavior. Qiu et al. [15] proposed a distributed UAV swarm algorithm that uses bird flocking intelligence to guide a swarm of drones with multiple leading individuals to approach a target synchronously without collision. Cao et al. [16] established a coordinated control system for a group of four quadrotors based on the hierarchical mechanism of bird flocks, successfully achieving cooperative behaviors and formation functions among the aircraft. Huo et al. [17] proposed a pigeon-inspired circular formation control method based on intelligent behavior during hovering, solving the problem of cooperative circular formation with limited target information for unmanned aerial vehicle (UAV) swarms. Li et al. [18] proposed a path-planning method based on pigeon obstacle-avoidance behavior and applied it to UAV swarm path-planning problems, allowing the UAV swarm to make real-time path decisions based on the obstacle position and successfully avoid obstacles under external interference. Ding et al. [19] were inspired by the information transmission mechanism between individuals in bird flocks and proposed a method for constructing an interactive topology of a UAV swarm, which ensures the swarm’s formation stability and system scalability at all times.

In fact, previous studies on collective behavior in birds have focused more on the interaction mechanisms and leadership networks under average individual behavior. However, in more specific scenarios, such as sudden turns or zigzags, the leader–follower structure and collective decision-making mechanisms of the group are still unclear. Therefore, developing a more complex dynamic model to vividly describe the motions of pigeon flocks is an urgent task. Previous studies [13,14,20,21] have widely used velocity correlation functions between pairs of pigeons as a starting point to measure the social organization structure of pigeon flocks. However, compared to velocity, acceleration better reflects the individual’s motion trend during trajectory fluctuations. Hence, an “acceleration alignment function” is proposed to better understand the dynamic leadership behavior between individuals and reflect the leader–follower relationship of the group under sudden conditions. And the leader–follower structure is linked to the positions of individual members in the turning direction. The goal is to reveal the potential factors influencing the decision-making mechanism of pigeon flock leadership and refine the collective decision-making mechanism in special circumstances. More specifically, a dynamic leadership mechanism will be revealed based on the evolution of acceleration in the pigeon flock during zigzag motion, which is suitable for describing the leader–follower structure and leader decision-making mechanism exhibited by the flock in turbulent trajectory segments.

By studying biological collectives, insights can be gained into the interaction patterns, information transmission mechanisms, leadership, and decision-making among the individuals of the group. These patterns can be utilized to design and optimize the topology, individual behavior rules, and information exchange mechanisms of swarm intelligence algorithms. By uncovering the behavioral patterns exhibited by flocks in specific situations, new ideas, and inspirations can be provided for an important distributed control strategy in the field of robot swarms: the leader–follower method. This is especially relevant when robot swarms encounter environmental changes, determining whether and how to change leaders.

The rest of the paper is organized as follows. Section 2 introduces the methods for data analysis and defines indicators to describe the internal leadership mechanism within a flock. Section 3 analyzes homing flight data in flocks, demonstrating the leader–follower structure and collective decision-making mechanisms during sudden turns or zigzag movements. The discussion of the results is presented in Section 4. Conclusions are drawn in Section 5.

## 2. Materials and Methods

### 2.1. Data Acquisition and Description

The three sets of pigeon homing data used in this paper were obtained from the experiment conducted by Nagy et al. [9], involving 10 pigeons (labelled A to M) aged between 1 and 5 years. These experiments received permission and support from the Hungarian Pigeon Racing Federation. All the pigeons had prior experience with homing, and before the experiment, each pigeon was outfitted with a simulated device made of plastic clay weighing 16 g, identical in size and weight to the GPS logger. The GPS devices recorded data points, including latitude, longitude, altitude coordinates, and time, with a time resolution of 0.2 s. To address instances of GPS signal loss, the interpolation of missing data points was performed by averaging the positions of neighboring recorded locations. Due to the 0.2 s sampling interval of the GPS data, the time unit mentioned in this paper is 0.2 s.

### 2.2. The Measurement of Flock State

#### 2.2.1. Flight Status and Grouping Criteria

A previous study by Zaitouny et al. [22] revealed that pigeons typically exhibit several different states during the flight process, including two distinct periods of “flight and non-flight” as well as atypical behaviors such as scattering. Therefore, within a single group of homing flights, the composition of the pigeon flock may vary across different time intervals. Furthermore, considering the research focus on analyzing the homing flight periods of the pigeon flock, it is necessary to exclude non-flight time intervals of the entire flock. The flight status of the pigeon flock can be expressed as follows:

(1) $$I(t)=\left\{\begin{array}{ll} 1, & \mathrm{if}\;\frac{1}{n}\sum_{i=1}^{n}\left||v_{i}(t)|\right|\geq 2\\ 0, & \mathrm{otherwise}\; \end{array}\right.$$

where *I*(*t*) represents the flight status of the pigeon flock at time *t*, *n* is the number of flock members, and the magnitude of an individual i’s velocity vector, i.e., the Euclidean norm, is denoted as ‖***v****_i_*(*t*)‖. If the average speed of all *n* pigeons in the flock is greater than or equal to 2 m/s, it indicates that the flock is flying; otherwise, the flock is not flying. The value of 2 m/s is used as a threshold for determining whether the flock is flying, which is chosen based on previous studies and observations of pigeon flight behavior.

In the homing flight, the trajectories of homing pigeons are nearly linear, unlike the circular trajectory observed in free-flying pigeons. Using a set of coordinates (x, y, z) for a group of pigeons as an illustration, the range of variation along the x-axis during the flight period varies from approximately 2900 to 9100 m, along the y-axis from 2000 to 14,000 m, and the z-axis from 100 to 230 m. Hence, it is reasonable to assume that the movement of pigeons during flight predominantly occurs within the xy-plane, with minimal motion observed in the z-direction. Additionally, due to the presence of atypical behavior, such as outliers among the pigeons, the grouping criteria for different periods of flock composition can be defined as follows:

(2) $$G(t)={\left\{p_{i}\right\}}_{i=1}^{m},\forall i,j\in \{1,2,\ldots ,m\},||p_{i}-p_{j}||<d$$

where *G*(*t*) represents a pigeon flock at time *t*, a set consisting of multiple members. The xy coordinates of each member are denoted as ***p****_i_*, and the value of *m* represents the total number of members in the flock. The Euclidean distance, ║***p****_i_*(*t*)−***p****_j_*(*t*)║, is used to measure the spatial distance between two members. The distance threshold *d* is used to determine whether a group of pigeons can form a flock. By comparing the Euclidean distance with the predefined distance threshold *d*, if the distance is smaller than *d*, the members ***p****_i_* and ***p****_j_* are categorized as belonging to the same period and group.

#### 2.2.2. Quantification of Flock Synchronization

When pigeons start flying or landing, their trajectory along the z-axis undergoes abrupt changes. At this time, the direction of movement among members within the flock is relatively chaotic. However, the primary focus is on the period when a highly ordered state emerges during a pigeon’s homing flight. To assess the level of orderliness in the flock, high-resolution and lightweight GPS devices can be utilized to obtain rich velocity information within the flock and correlate it with the velocity vectors. The order parameter [23] can be defined as follows:

(3) $$\phi (t)=\frac{1}{m}\left\|\sum_{i=1}^{m}\frac{v_{i}(t)}{\left||v_{i}(t)|\right|}\right\|$$

where the order parameter *ϕ* represents the level of organization in a flock and ***v****_i_*(*t*) represents the velocity vector of pigeon *i* during its homing flight. Apparently, complete disorder corresponds to the index *ϕ* being zero, while complete synchrony corresponds to the index *ϕ* being one.

To accurately measure the degree of velocity direction synchronization among individual pigeons during a period of steady flight, their synchronization status can be evaluated by calculating the difference in angle between velocity vectors at a given moment. The velocity direction consistency [24] can be defined as follows:

(4) $$\psi_{i}(t)=\frac{1}{\left|G(t)\right|}\sum_{j\in G(t)}\frac{\left\|v_{i}(t)-v_{j}(t)\right\|}{\left\|v_{i}(t)\right\|}$$

where *ψ_i_*(*t*) represents the velocity direction consistency of a flock. The ***v****_i_*(*t*) and ***v****_j_*(*t*) represent the velocity vectors of pigeon *i* and *j* in the flock at time *t*; ║***v****_i_*(*t*)║ denotes the magnitude of *i*’s velocity vector, ║***v****_i_*(*t*) − ***v****_j_*(*t*)║ denotes the magnitude of the difference between ***v****_i_*(*t*) and ***v****_j_*(*t*). |*G*(*t*)| represents the number of pigeon members. A lower value of *ψ_i_*(*t*) indicates that pigeon *i* and other pigeons are well synchronized with respect to velocity direction, whereas a higher value indicates less synchronization.

During homing flight, pigeon flocks do not move at a constant speed. There are moments when they change direction, and in such instances, acceleration reveals the motion trend of the flock better than velocity. Therefore, the degree of individual acceleration synchronization can be defined as follows:

(5) $$\theta_{i}(t)=\arccos\frac{{\hat{a}}_{i}}{{\hat{a}}_{g}}$$

where *θ_i_*(*t*) represents the degree of acceleration synchronization between individuals and the collective. ${\hat{a}}_{i}$ represents the unit acceleration vector of the pigeon *i*, and ${\hat{a}}_{g}$ represents the average unit acceleration vector of the flock, and ${\hat{a}}_{g}=\frac{1}{\left|G(t)\right|}\sum_{j=1}^{m}\frac{a_{j}}{\left||a_{j}|\right|}$.

### 2.3. Dynamic Leadership during Flock Direction Change

This section will introduce the study of characteristic indicators for flock direction changes. It is crucial to select appropriate indicators to analyze and quantify social behaviors and leadership structures of pigeon flocks in different flight states. During the homing flight of pigeon flocks, the trajectories are generally smooth most of the time, but there are also periods when the flock clearly changes direction. Therefore, it is necessary to further explore how to utilize the rich acceleration and velocity information from GPS data to reflect the changes in leadership among different flock members. In addition, the first step is to characterize the flight trajectory of the flock, using a numerical approximation method to estimate the curvature [25]:

(6) $$k_{i}(t)=\frac{\sqrt{{{\ddot{x}}_{i}}^{2}+{{\ddot{y}}_{i}}^{2}+{{\ddot{z}}_{i}}^{2}}}{{\left({\dot{x}}_{i}^{2}+{\dot{y}}_{i}^{2}+{\dot{z}}_{i}^{2}\right)}^{3/2}}$$

where *k_i_*(*t*) represents the curvature of pigeon *i* at time *t*, ${\ddot{x}}_{i}$, ${\ddot{y}}_{i}$, ${\ddot{z}}_{i}$ denote the second derivative of the *x*, *y*, and *z* coordinate sequences of pigeon *i* and can be calculated using difference approximation; ${\dot{x}}_{i}$, ${\dot{y}}_{i}$, ${\dot{z}}_{i}$ denote the first derivative, which can be calculated by the same method.

The curvature of the whole *k_g_*(*t*) can be defined as follows:

(7) $$k_{g}(t)=\frac{1}{\left|G(t)\right|}\sum_{j\in G(t)}k_{j}(t)$$

To investigate the correlation between the motion trend (represented by the curvature approximated by numerical methods) and velocity or acceleration information during flock turning, the Pearson correlation coefficient [26] and Spearman correlation coefficient [26] are introduced. The former is used to characterize linear correlations, while the latter is used to characterize nonlinear correlations. Additionally, this approach can also demonstrate the validity of using difference approximations to calculate curvature in Equation (6). The Pearson correlation coefficient can be given as follows:

(8) $$\rho_{X_{a_{g}},Y_{k_{g}}}=\frac{\mathrm{cov}(X_{a_{g}},Y_{k_{g}})}{\sigma_{X_{a_{g}}}\sigma_{Y_{k_{g}}}}=\frac{E\left[\left(X_{a_{g}}-\mu_{X_{a_{g}}}\right)\left(Y_{k_{g}}-\mu_{X_{k_{g}}}\right)\right]}{\sigma_{X_{a_{g}}}\sigma_{Y_{k_{g}}}}$$

Equation (8) represents the Pearson correlation coefficient between the norm of the acceleration of a flock of pigeons and its curvature. Specifically, $\rho_{X_{a_{g}},Y_{k_{g}}}$ denotes the Pearson correlation coefficient between the norm of acceleration *a_g_* and the curvature *k_g_* of the flock. $\mathrm{cov}(X_{a_{g}},Y_{k_{g}})$ is the covariance between *a_g_* and *k_g_*, and $\sigma_{X_{a_{g}}}$ and $\sigma_{Y_{a_{g}}}$ denote the standard deviation of *a_g_* and *k_g_*, respectively. The Pearson correlation coefficient between the norm of velocity and the curvature of the flock $\rho_{X_{v_{g}},Y_{k_{g}}}$ can be calculated similarly to Equation (8), where *v_g_* denotes the norm of velocity of the flock.

The Spearman correlation coefficient [26] between the norm of acceleration *a_g_* and the curvature *k_g_* of the flock $K_{v_{g}k_{g}}$ can be given as follows:

(9) $$K_{a_{g}k_{g}}=1-\frac{6\cdot \sum_{i=1}^{N}{\left(\mathrm{rank}(a_{gi})-\mathrm{rank}(k_{gi})\right)}^{2}}{N\cdot (N^{2}-1)}$$

where $\mathrm{rank}(a_{gi})-\mathrm{rank}(k_{gi})$ denotes the rank difference between the *i*-th pair of *a_g_* and *k_g_*. *N* represents the number of the *a_g_* or *k_g_*. The Spearman correlation coefficient between the norm of velocity and the curvature of the flock $K_{v_{g}k_{g}}$ can be calculated similarly to Equation (9).

Turning behavior involves a transition from one direction to another. While velocity only provides information about an object’s spatial movement, acceleration captures the dynamic motion process more effectively. Furthermore, changes in acceleration can reflect the flock’s behavioral intent. When the flock is preparing to turn, acceleration will change. Through the analysis of acceleration data, inferences can be made regarding whether the flock is preparing for or on the verge of executing a turning action, as well as the potential direction of turning. Therefore, a metric is defined here to infer the changes in leadership between a pair of pigeons when the flock is turning, which can be defined as follows:

(10) $$D_{ij}(t,\tau )=\frac{a_{i}(t)}{\left||a_{i}(t)|\right|}\cdot \frac{a_{j}(t+\tau )}{\left||a_{j}(t+\tau )|\right|}$$

Equation (10) represents a metric, denoted as $D_{ij}(t,\tau )$, with parameters of time *t* and delay time *τ*. The $D_{ij}(t,\tau )\in [-1,1]$; this scalar value represents the degree of similarity in the acceleration directions between pigeon *i* at time *t* and pigeon *j* at time *t* + *τ*. The value will be close to 1 if their acceleration directions are aligned. If their acceleration directions differ significantly, the value will be close to −1. τ∈[−2,2], which means that the focus is on the similarity in acceleration directions between pairs of pigeons within a time interval of 4 s.

By calculating $D_{ij}(t,\tau )$ at different delay times, the optimal time ${\overset{⌢}{\tau }}_{ij}$ can be determined for matching their acceleration directions. This ${\overset{⌢}{\tau }}_{ij}$ value represents the best coordinated time between them, where their acceleration directions exhibit the highest similarity or alignment, and can be expressed as follows:

(11) $${\overset{⌢}{\tau }}_{ij}=\max_{\tau }D_{ij}(t,\tau )$$

It is to be noted that, in this case, the $D_{ij}(t,{\overset{⌢}{\tau }}_{ij})$ for a pair of pigeons must be greater or equal to 0.96. This requirement ensures that the detected similarity in acceleration directions between the pair is substantial rather than being due to random fluctuations or noise. It is obvious that positive ${\overset{⌢}{\tau }}_{ij}$ means pigeon *i* is leading pigeon *j*, while negative ${\overset{⌢}{\tau }}_{ij}$ means pigeon *j* is leading pigeon *i*.

To quantify the leadership status of a pigeon within the flock at time *t*, an indicator can be defined as follows:

(12) $$l_{i}(t)=\sum_{j\in G(t),j\neq i}L({\overset{⌢}{\tau }}_{ij})$$

(13) $$L({\overset{⌢}{\tau }}_{ij})=\left\{\begin{array}{ll} 1, & \mathrm{if}\;{\overset{⌢}{\tau }}_{ij}\geq 0\\ 0, & \mathrm{otherwise}\; \end{array}\right.$$

where *l_i_*(*t*) represents the dominance of pigeon *i* over other members of the flock at time *t*. A higher value of *l_i_*(*t*) implies that pigeon *i* has a greater leadership influence within the flock, while a lower value suggests that pigeon *i* is more influenced by others.

## 3. Results

### 3.1. The Flight Status and Composition of the Pigeon Flock

Flight is one of the important behaviors in pigeon flocks, which is highly collective and coordinated behavior by multiple pigeons. Observing the flight status of the pigeon flock, including flight speed, direction, and flock formation, can provide important information on decision making and coordination within the flock. On the other hand, when the pigeons are not flying, they exhibit different behaviors, such as foraging and resting, which may cause unnecessary interference in data analysis. Therefore, it is important to classify the flight status of the pigeon group accurately, and the method described in Section 2.2.1 can be used to ensure the accuracy of the data.

**Figure 1:**
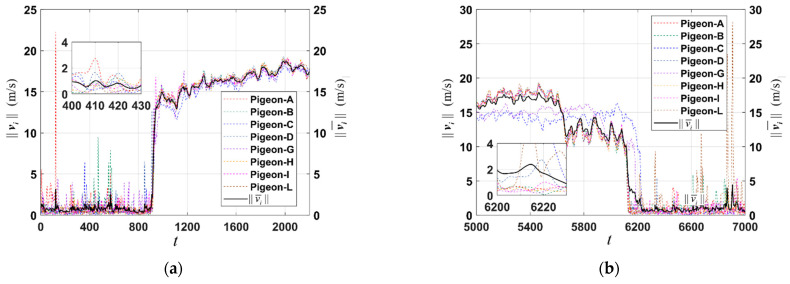
The flight status of the pigeon flock (A, B, C, D, G, H, I, L) during different phases of homing flight. (**a**) The flight status of the pigeon flock during the initial phase of homing flight. (**b**) The flight status of the pigeon flock during the final phase of homing flight.

Different colored lines represent the speeds corresponding to pigeons with different IDs, while the black line represents the average speed of the entire flock. The zoomed-in section shows a specific period during the non-flight phase. In Figure 1a, it is evident that during the initial phase of homing flight, there is a clear distinction between the flying and non-flying periods of the pigeon flock. Between *t* = 0−900, the average velocity of the flock remains below 2 m/s. During this period, the calculated indicator function *I*(*t*) is 0, indicating that the flock is non-flying. However, after surpassing *t* = 900, the flock enters the flight phase, and the average velocity increases significantly. Consequently, *I*(*t*) changes to 1, indicating that the flock is in flight. As shown in Figure 1b, towards the later phase of homing flight, around *t* = 6100, there is a turning point where the flock transitions from a flight phase to a non-flight phase. At this stage, most pigeons have already returned home, and almost all pigeons maintain a speed below 5 m/s. Consequently, *I*(*t*) is calculated as 0 during this period. This turning point demonstrates a shift in the behavior of the flock as it exits the flight phase and enters the homing phase.

The main focus of this study is the analysis of leader decision-making information during the flight phase of pigeon flocks. In subsequent analyses, a specific emphasis will be placed on exploring relevant information during the flight phase.

**Figure 2:**
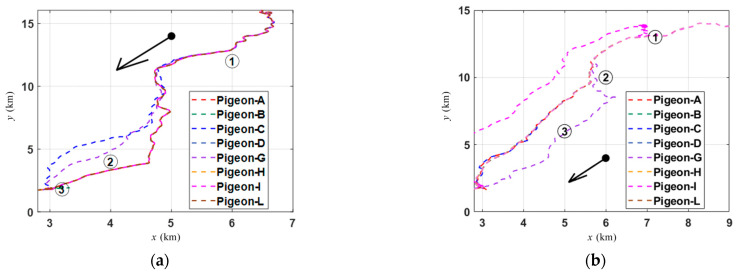
The grouping patterns exhibited by the flight trajectories of two pigeon flocks on the xy- plane. (**a**) Grouping of the pigeon flock (A, B, C, D, G, H, I, L). (**b**) Grouping of the pigeon flock (A, C, D, F, G, H, I, J, K, L).

To investigate the changes in pigeon group members during homing flight, the flight trajectories of two pigeon flocks on the xy-plane are illustrated in Figure 2. In Figure 2a, the members undergo three significant stages of change. Some individuals suddenly separate from the smooth cluster flight, while others rejoin the group during homing. Similar behavior is also observed in Figure 2b. By making these observations, a better understanding can be gained regarding the dynamic changes in the composition of pigeon groups during homing flight.

The different colored lines refer to the xy coordinates of pigeons with different IDs, while black arrows indicate the overall direction of the homing flight of the pigeon flock, and the sequence numbers represent the timing of different groupings. Data in Table 1 are based on Equation (2), where the threshold *d* is set to 20 m. Only pigeons with distances less than *d* are considered to belong to the same flock. The table lists the status of two flocks during the return journey. It was found that their memberships were not fixed at different time points. However, the memberships of some flocks remained unchanged during certain periods, which is an important period for subsequent analysis. It should be noted that only the situation of two flocks is listed in the table, but in fact, Equation (2) applies to any number of pigeons.

### 3.2. Measurement of Flock Synchronization

**Figure 3:**
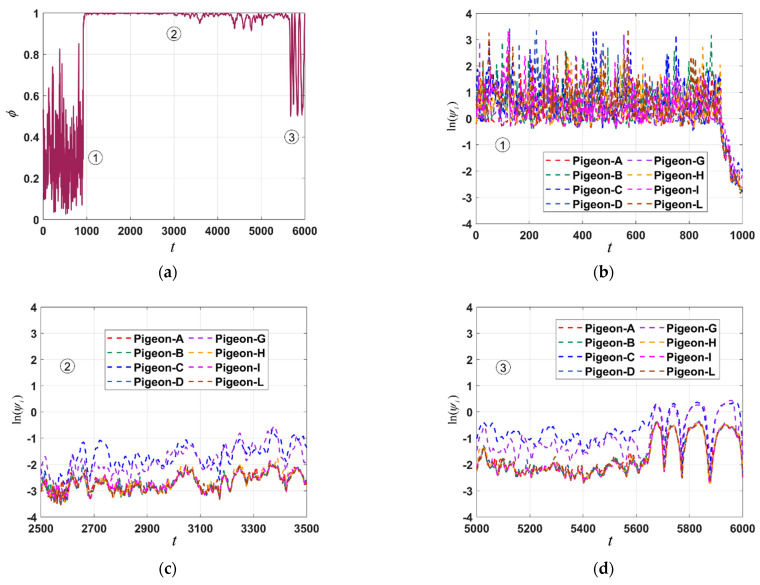
The collective and individual velocity orderliness exhibited by the pigeon flock at different stages. (**a**) Collective orderliness of the pigeon flock (A, B, C, D, G, H, I, L). (**b**–**d**) Velocity direction consistency of the pigeon flock (A, B, C, D, G, H, I, L).

The degree of collective orderliness of the pigeon flock at different time points can be revealed by the flock’s orderliness graph (Figure 3a), which is obtained using Equation (3) in Section 2. The time indices ①, ②, ③ represent periods of significant fluctuations in collective orderliness. The graph facilitates the evaluation of collective behavioral patterns, dynamic changes in flock structure, and the group’s stability. In Figure 3a, noticeable variations in the degree of orderliness are observed across different periods. Initially, during the early phase of the homing flight, the flock exhibits a disordered state, gradually transitioning to a state of organized flight. 

In Figure 3b–d, the time indices ①, ②, ③ in the top left corner represents the velocity direction consistency of different individuals during the corresponding periods in Figure 3a. The vertical axis of the figures represents the natural logarithm of the velocity direction consistency. This is calculated to ensure that the figures are consistently presented on the same scale, allowing for a more intuitive comparison. In Figure 3b, by computing the velocity direction consistency metric from Equation (4) in Section 2, it can be observed that during the initial phase of the homing flight, the eight individuals comprising the pigeon flock exhibit very poor consistency in their velocity directions, indicating a state of complete disorder (*ψ_i_*(0–1000) ≥ 10). However, in Figure 3c, it is evident that the flock has entered a state of velocity synchronization, indicating a relatively smooth flight (*ψ_i_*(2500–35,000) ≤ 0.5). However, it is worth noting that when *t* ≥ 3100, Pigeon-C and Pigeon-G start showing a trend of inconsistent velocity directions, and in Figure 3d, this trend becomes more pronounced. Interestingly, this phenomenon corresponds to the grouping observed in Figure 2a.

### 3.3. Dynamic Leadership during Turns in Pigeon Flock

**Figure 4:**
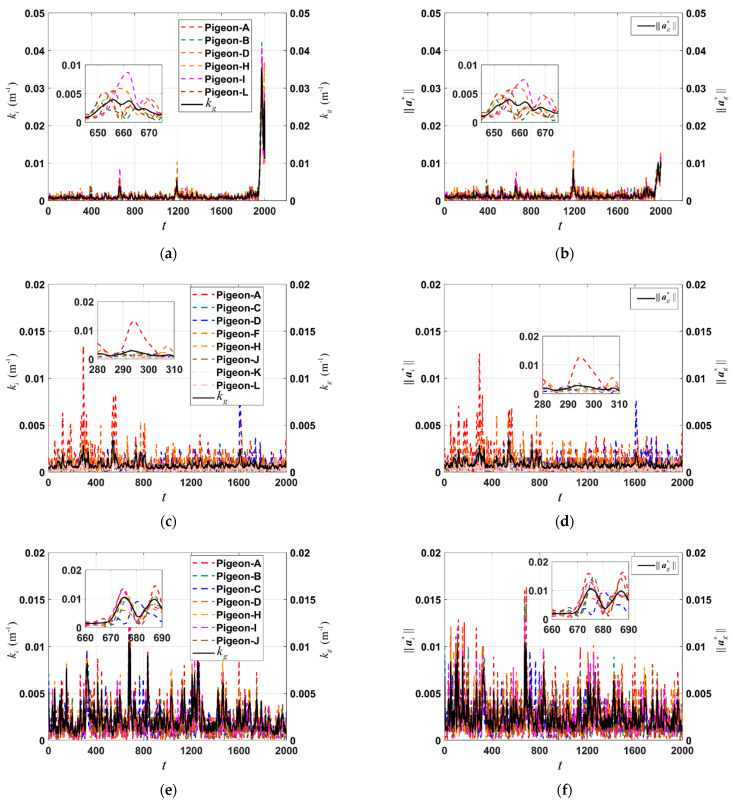
The trajectory curvatures (**a**,**c**,**e**) and corresponding accelerations (**b**,**d**,**f**) during the or-gnized flight segment of the flock (A, B, C, D, G, H, I, J), flock (A, C, D, F, G, H, I, J, K, L) and flock (A, B, C, D, G, H, I, J, L).

After grouping, flight segment identification, orderliness analysis, and direction consistency analysis of three homing pigeon flocks, three sets of images (Figure 4) are obtained. The organized flight segment is extracted from the overall flight process. Therefore, the x-axis in the graph starts at 0. The legend represents the individuals that comprise the flock. In figures b, d, f, $\left||a_{i}|\right|$ and $\left||a_{g}|\right|$ represent the magnitudes of individual acceleration and flock acceleration, respectively. To observe the relationship more intuitively between acceleration magnitude and flight trajectory curvature, it is necessary to align the two on the same scale. Therefore, a scale factor related to flight trajectory curvature is multiplied to $\left||a_{i}|\right|$ and $\left||a_{g}|\right|$ to obtain $\left||a_{i}^{*}|\right|$ and $\left||a_{g}^{*}|\right|$. In Figure 4a, the members of the original flock (A, B, C, D, G, H, I, J) during the organized flight segment were Pigeon-A, Pigeon-B, Pigeon-D, Pigeon-H, Pigeon-I and Pigeon-J. Their corresponding trajectory curvatures exhibit significant peaks at *t* = 650, 1190, 1980, indicating a tendency for flock reorientation during these moments. This phenomenon is further illustrated in the zoomed-in portion of Figure 4a. Correspondingly, Figure 4b depicts the acceleration variations at the corresponding time points. Interestingly, the occurrence of acceleration peaks aligns reasonably well with the timing of curvature peaks. This phenomenon is also observed in the organized flight segments of the other two homing pigeon flocks.

Drawing upon the data presented in Table 2, the following conclusions can be made. Among the three pigeon flocks, a notable positive linear correlation exists between acceleration and curvature, with the second flock exhibiting the strongest correlation. However, the Spearman correlation coefficient indicates a weak nonlinear relationship between acceleration and curvature. Both the linear and nonlinear relationships between velocity and curvature are also weakly correlated. In summary, when the acceleration of the pigeon group increases or decreases, the curvature will correspondingly increase or decrease. This strong correlation reveals a close association between acceleration and curvature, indicating the important role of acceleration in adjusting the curvature of the flight path in pigeon flocks, especially when the trajectories of pigeon flocks exhibit significant fluctuations.

**Figure 5:**
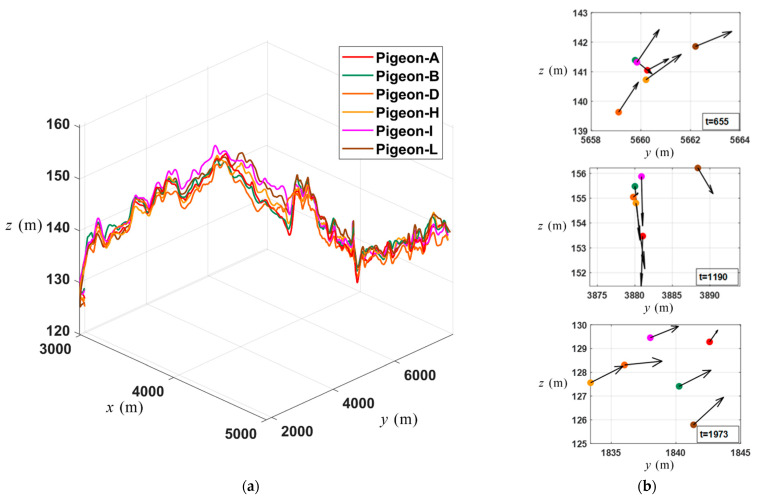
(**a**) The three-dimensional flight trajectory of the flock (A, B, D, H, I, L) during coordinated flight segment. (**b**) The acceleration vectors (yz-plane) corresponding to Pigeon-A, Pigeon-B, Pigeon-D, Pigeon-H, Pigeon-I, and Pigeon-L at three moments of turning.

In Figure 5a, the range of variation in the flight trajectory along the z-axis is much smaller than that along the x-axis and y-axis. Therefore, to better describe the motion changes along the z-axis, the three coordinate axes are scaled differently. The flight of the flock in the xy-plane is relatively stable, while it fluctuates in the z-direction, suggesting that the turns during the flight mainly occur in the z-direction. This phenomenon can be observed in all three sets of homing flights.

Based on the previously analyzed strong positive correlation between curvature and acceleration, the times (*t* = 655, 1190, 1973) when curvature peaks appeared in Figure 4a are referred to, and the accelerations (yz-plane) of different individuals at the corresponding moments are visualized in Figure 5b. The solid dots of different colors represent the coordinates of different pigeons in the yz-plane. The yz-plane was chosen because Figure 5a indicates that turns during flight primarily occur in the z-direction, allowing for a more intuitive observation of the acceleration evolution of individuals during turning. It is observed that when the flock undergoes sudden turns, the accelerations of the majority of individuals show consistent trends, which may be due to the operation of certain leadership mechanisms. However, the leadership mechanisms reflected by this trend still require further in-depth analysis to reveal the underlying dynamics and patterns driving flock-turning behavior. Through the observation of Figure 5a,b, further exploration can be conducted to decipher the reasons behind the turns that take place during the flight of pigeon flocks, as well as the potential existence of dynamic leadership mechanisms.

**Figure 6:**
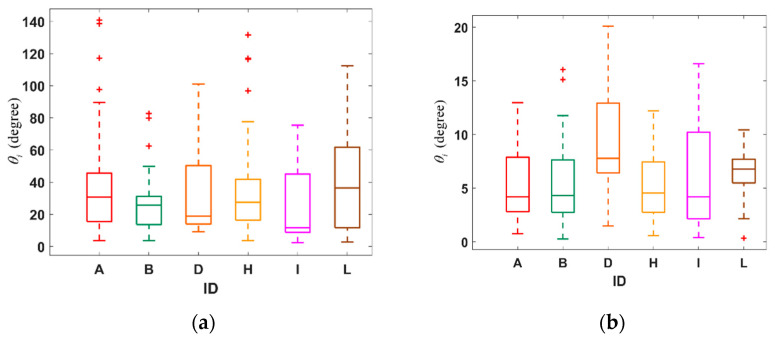
(**a**) The boxplot of relative acceleration angle changes of pigeon flock members at *t* = 655 (within 6 s). (**b**) The boxplot of relative acceleration angle changes of pigeon flock members at *t* = 1973 (within 6 s).

In Figure 6b, when there is a tendency for the pigeon flock to change direction in the z-axis, the differences in acceleration angles among the flock members within six seconds are generally within 20 degrees, which indicates that the pigeon flock members demonstrate a certain level of coordination and synchronicity in their acceleration when making turns. The relatively small range of angle differences suggests that this behavior may be a collective response to external stimuli or internal communication mechanisms, enabling the flock to maintain cohesion and adjust their flight trajectory. The observed consistency in acceleration angles implies the existence of cooperative behavior or leadership mechanisms guiding the flock’s turning movements.

Moreover, a similar phenomenon also exists in Figure 6a. However, in Figure 6a, it can be observed from the box plots that at certain moments, Pigeon-A and Pigeon-H exhibit larger outliers in their *θ_i_*, which could be due to atypical behavior of the individual, but overall, the differences in angle between individual acceleration and group acceleration remain within a relatively small range.

**Figure 7:**
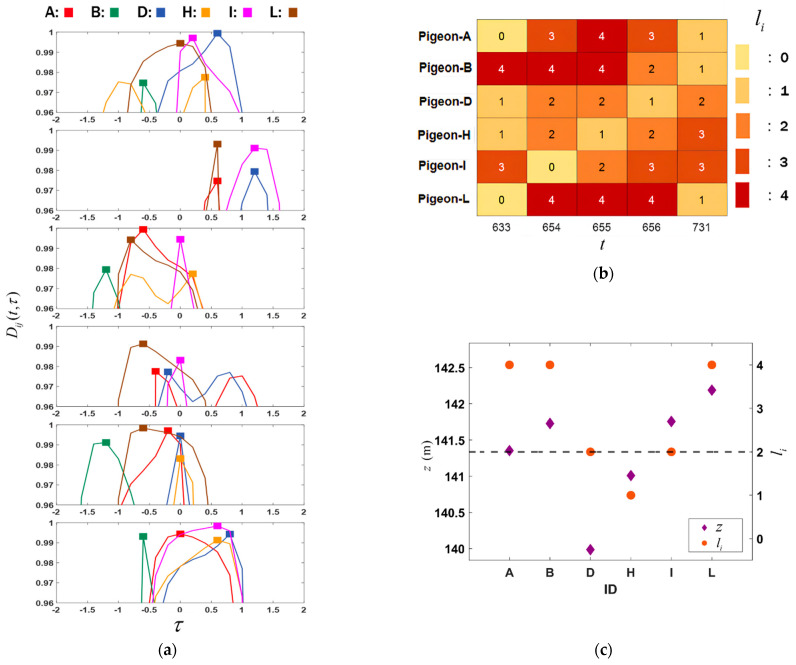
(**a**) The acceleration alignment function *D_ij_*(*t*,*τ*) during the turn at *t* = 655. (**b**) The heatmap of leadership function *l_i_*(*t*) at *t* = 633, 654, 655, 656 and 731. (**c**) Correlation between leadership fun-ction *l_i_*(*t*) and z-coordinate.

In Equation (10) of Section 2, the acceleration alignment function *D_ij_*(*t*,*τ*) is defined to infer the changes in leadership between a pair of pigeons when the flock is turning. Due to the short duration of turning behavior, a time delay interval *τ_ij_* ranging from −2 to 2 is set for analysis. Figure 6 indicates that acceleration exhibits higher coordination and synchronization during turning. Therefore, a threshold of 0.96 is set as $D_{ij}(t,{\overset{⌢}{\tau }}_{ij})$. In Figure 7a, the solid symbols indicate the optimal acceleration matching time, and internal dynamic leadership relationships within the flock during turning can be observed. Taking Pigeon-B as an example, its ${\overset{⌢}{\tau }}_{BA}=0.6$, ${\overset{⌢}{\tau }}_{BD}=1.2$, ${\overset{⌢}{\tau }}_{BI}=1.2$ and ${\overset{⌢}{\tau }}_{BL}=0.6$, which implies that at *t* = 655, Pigeon-B exhibits leadership over Pigeon-A, Pigeon-D, Pigeon-I, and Pigeon-L, and the magnitude of its leadership can be reflected by the value of ${\overset{⌢}{\tau }}_{ij}$.

In Figure 7b, a selection of five time points (*t* = 633, 654, 655, 656, 731) is selected. Among them, *t* = 654, 655, and 656 represent the moments when the pigeon flock changes direction, while *t* = 633, 731 indicates relatively stable curvatures before and after the turning. The purpose is to obtain the leadership organizational structure of the pigeon flock during the turning and when they are not changing direction. Taking *t* = 655 as an example, it can be observed that *l_A_*(655) = 4, *l_B_*(655) = 4, *l_D_*(655) = 2, *l_H_*(655) = 1, *l_I_*(655) = 2 and *l_L_*(655) = 4, which implies that Pigeon-A, Pigeon-B and Pigeon-L with larger *l_i_*(*t*) values have higher leadership positions during the turning, while other members of the flock are influenced by these three individuals and assume follower roles. Similarly, the flock does not exhibit directional change at *t* = 633; Pigeon-A and Pigeon-B hold higher leadership positions, while Pigeon-L becomes a follower.

Figure 7c presents an intuitive display of the internal leadership structure of a pigeon flock during turning, along with the associated factors. The grey dashed line represents the center position of the flock in the z-direction. It can be found that at *t* = 655, Pigeon-L, which was on the outside of the flock, occupied a leadership position, while Pigeon-A and Pigeon-B were closer to the center of the flock, which means that the individuals on the outside of the flock participated in the leadership process during the turning phase and demonstrated strong leadership. It is worth noting that some individuals seem to be in a leadership position regardless of whether the flock turns or not, such as Pigeon-A and Pigeon-B. However, when the flock does not turn, individuals on the outside of the flock are more likely to be in the following position. For example, Pigeon-L lost its leadership position at *t* = 633, 731.

## 4. Discussion

The leadership system exists in many species in nature, such as elephants [27], sheep [28], and pigeons [9]. Prior research [29] has demonstrated that in larger groups, there is a pervasive correlation among members, and hierarchical networks can be formed based on individual motivations, navigational knowledge, abilities, etc. A previous study [13] found that interactions correlate with the strength of alignment measured by fluctuations in speed; i.e., whether a pair of pigeons exhibits information exchange behavior can be assessed through the fluctuation of velocity information, suggesting an intermittent alignment mechanism within the flock. Paper [21] also indicated that the fastest-flying individuals eventually take the lead, where they can exert more significant influence on the flock’s movement. However, previous studies on collective behavior in birds have mainly focused on the mechanisms of interaction and leadership networks under average individual behavior, while there is still considerable uncertainty about the leader–follower structure and collective decision-making mechanisms of groups, particularly in specific scenarios such as sudden turns or zigzag movements.

This paper is based on research on the homing flight movement of pigeon flocks and infers different leadership strategies adopted by the flock in different flight stages according to the acceleration information [30,31] of specific scenarios. It presents a dynamic leadership mechanism during the homing flight of pigeon flocks, where the curvature of flight trajectories and the linear and nonlinear coefficients of acceleration and velocity are compared to understand that acceleration more significantly represents the flock’s movement trend when curvature exhibits larger fluctuations. The leadership relationship and the magnitude of leadership are characterized by defining the alignment function of acceleration *D_ij_*(*t*,*τ*), and the leadership position of individuals within the flock is represented by Equations (12) and (13). The results in Figure 7 demonstrate the changes in flock acceleration consistency during turning moments, highlighting the leadership relationships within the flock and the changes in individual leadership positions in different flight stages. Associating this leadership position with the sequential order in the z-direction of the flock indicates that the flock undergoes leader transitions in different flight states, where individuals on the outer side of the flock have significantly increased leadership weight along the direction of the flock movement during turns but lose their original leadership position as the turning trend disappears.

This paper holds certain value and potential in both theory and practice. It contributes to a deeper understanding of the mechanisms and patterns of collective intelligence revealed through collective behavior, which is significant for the research and application of collective intelligence. It can provide insights and references for the control and coordinated decision-making of multiple robots in emergencies. However, there are limitations in this study. It is based on the homing flight of pigeons, and further exploration is needed to determine its applicability to other species or scenarios. More in-depth quantitative analysis and experimental validation are required to refine the theoretical models and methods further and address practical control issues in multi-robot coordination.

## 5. Conclusions

This paper investigates the leader–follower structure and collective decision-making mechanism in pigeon flocks during sudden turns or zigzag movements. Through observation and analysis of homing flight in pigeon flocks, an “acceleration alignment function” is proposed to understand the dynamic leadership behavior between individuals and to establish a more complex dynamic model for describing flock motion by linking leader–follower relationships with individual positions. The paper reveals the leadership mechanisms within the flock in specific scenarios. Analysis results indicate that the flock is led by a fixed leader during stable flight, while external individuals are not considered leaders. However, when the flock abruptly changes direction, individuals on the periphery of the group transition from followers to leaders, while the original leader maintains its leadership position. This dynamic leadership mechanism enables the flock to maintain flexibility and stability under different flight conditions. In the future, our work will focus on applying this dynamic leadership mechanism to swarm control and collaborative decision-making in multi-robot coordination, particularly in complex environments and emergencies.

## Figures and Tables

**Table 1:** Grouping status corresponding to different time points.

Time	①	②	③
**Flock1**	{A, B, C, D, G, H, I, L}	{A, B, D, H, I, L }	{A, B, C, D, G, H, I, L}
**Flock2**	{A, C, D, F, G, H, I, J, K, L}	{A, C, D, F, G, H, J, K, L}	{A, C, D, F, H, J, K, L}

**Table 2:** Correlation between curvature and acceleration/velocity for the three flocks.

Flock	Flock (A, B, C, D, G, H, I, J)	Flock (A, C, D, F, G, H, I, J, K, L)	Flock (A, B, C, D, G, H, I, J, L)
$\rho_{a_{g}k_{g}}$	0.8234	0.9709	0.9030
$\kappa_{a_{g}k_{g}}$	−0.0530	0.0652	−0.0404
$\rho_{v_{g}k_{g}}$	−0.7620	−0.1464	−0.4149
$\kappa_{v_{g}k_{g}}$	−0.2868	−0.1391	−0.4388

## Data Availability

Data are contained within the article.
